# FoxO1: a novel insight into its molecular mechanisms in the regulation of skeletal muscle differentiation and fiber type specification

**DOI:** 10.18632/oncotarget.12891

**Published:** 2016-10-25

**Authors:** Meng Xu, Xiaoling Chen, Daiwen Chen, Bing Yu, Zhiqing Huang

**Affiliations:** ^1^ Key Laboratory for Animal Disease-Resistance Nutrition of China Ministry of Education, Institute of Animal Nutrition, Sichuan Agricultural University, Chengdu, Sichuan, P. R. China

**Keywords:** FoxO1, skeletal muscle, differentiation, fiber type specification, molecular mechanisms

## Abstract

FoxO1, a member of the forkhead transcription factor forkhead box protein O (FoxO) family, is predominantly expressed in most muscle types. FoxO1 is a key regulator of muscle growth, metabolism, cell proliferation and differentiation. In the past two decades, many researches have indicated that FoxO1 is a negative regulator of skeletal muscle differentiation while contrasting opinions consider that FoxO1 is crucial for myoblast fusion. FoxO1 is expressed much higher in fast twitch fiber enriched muscles than in slow muscles and is also closely related to muscle fiber type specification. In this review, we summarize the molecular mechanisms of FoxO1 in the regulation of skeletal muscle differentiation and fiber type specification.

## INTRODUCTION

Skeletal muscle is the most abundant tissue in mammal's body, constituting approximately 40-50% of the body mass. In addition to its primary role in posture and movement, skeletal muscle performs a number of critical functions, such as the regulation of energy and glucose metabolisms [1, 2]. Notably, the differentiation and fiber type composition of skeletal muscle are critical for these functions. During skeletal muscle development, myoblasts derived from the differentiation of myotome progress along the myogenic pathway through cellular proliferation, terminal differentiation and fusion into multinucleated myofibers [3]. The muscle specific transcription factors, MyoD and the myocyte enhancer factor 2 (MEF2) families, which proteins result in reprogramming expression of genes involved in muscle differentiation, govern the skeletal myogenesis [4, 5]. In addition, skeletal muscle is a highly contractile and dynamic tissue with striking plasticity. The composition of diverse muscle fiber subtypes defined by metabolic activity and specific myosin heavy chain (MyHC) isoforms is closely relevant with muscle plasticity and function. Although the fiber type specification varies between different species, on the basis of specific MyHC isoform expression, in mammal adult skeletal muscle fibers are prevalently classified as type I (slow oxidative muscle fibers), type IIa (fast oxidative muscle fibers), type IIx/d (intermediate muscle fibers), and type IIb (fast glycolytic muscle fibers) [6]. Both the skeletal muscle differentiation and fiber type composition are regulated in response to changes in environment, physical activity or pathological conditions [7]. Multiple transcription factors are involved in these two programs by effecting a change in expression of certain specific genes.

In mammals, transcription factors of the forkhead box protein O (FoxO) family consists of four members: FoxO1 (FKHR), FoxO3 (FKHRL1), FoxO4 (AFX), and FoxO6, of which proteins play considerable roles in a diverse sets of cellular physiological functions. As transcription factors, they can perform their functions through binding to downstream gene promoter or tethering to the target site by protein-protein interaction with other transcription factors [8]. FoxO family members have been shown to regulate various cellular functions, including proliferation, survival, cell cycle, metabolism, and muscle atrophy [9–12]. In addition, FoxO proteins have also been implicated in myoblast, preadipocyte, and endothelial cell differentiation [13]. FoxO1-null mice died during embryonic development, while FoxO3- or FoxO4-null mice could survive [14–17], indicating that FoxO1 may be the most important factor in mammal's life among this family. The ability of FoxO is largely dependent on posttranscriptional modifications [18], such as phosphorylation [19]. Phosphorylated FoxO1 would be excluded from nucleus and thus lost its capacity of binding to target regulatory elements [20]. Previous studies have revealed that FoxO1 provokes myotube fusion of primary mouse myoblasts [21, 22]. But more recent researches support FoxO1 as an inhibitor of muscle differentiation [9, 13, 19]. Moreover, FoxO1 is believed to play a role in skeletal muscle fiber type specification [23–26]. FoxO1 transgenic mice showed a significant decrease in skeletal muscle mass and impaired skeletal muscle function, accompanied with the reduced expression of slow fiber genes [23, 24]. Reciprocally, FoxO1 conditional deletion in the soleus muscle resulted in a reduction of slow fiber and an increase of fast fiber formation [25]. These findings present controversial roles of FoxO1 in muscle fiber type composition.

Here, we summarize the most recent advances on the roles of FoxO1 in the regulation of skeletal muscle differentiation and fiber type specification. The molecular mechanism responsible for FoxO1 regulation in these two processes will be detailed in this review.

## UPSTREAM REGULATION OF FOXO1 IN SKELETAL MUSCLE DIFFERENTIATION

**Figure 1:**
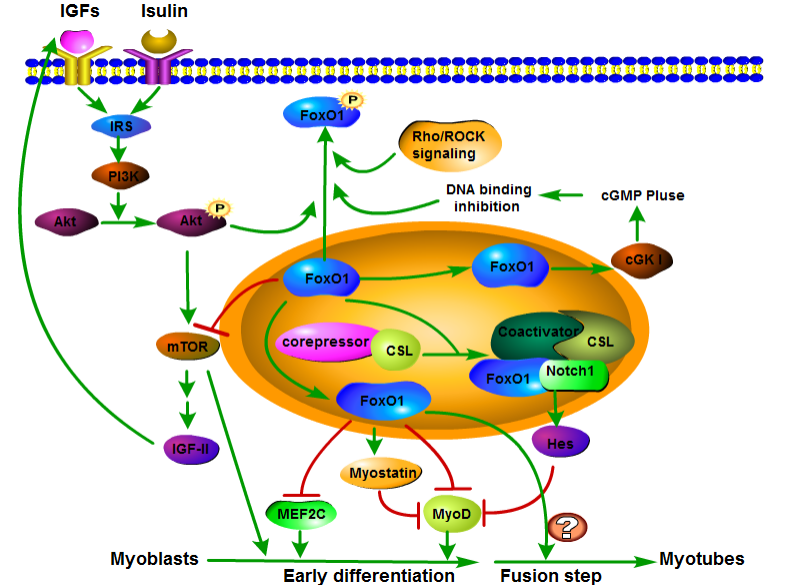
FoxO1 signaling pathway involved in skeletal muscle differentiation The FoxO1 upstream signals including IGFs, insulin and IRS regulate FoxO1 transcriptional activity through phosphorylating FoxO1 in a PI3K-Akt dependent manner. Phosphorylated FoxO1 will be excluded from nucleus and thus loses its capacity of binding to target regulatory elements. In addition, other signals, such as cGKI and Rho/ROCK signaling, directly mediate FoxO1 transcriptional activity by phosphorylation. Myostatin, MEF2C, MyoD and mTOR are downstream factors of FoxO1. FoxO1 negatively regulates myoblast early differentiation through promoting myostatin and inhibiting MEF2C, MyoD and mTOR. Then the decrease of MEF2C, MyoD and mTOR delays myoblast early differentiation. In addition, the relationship among FoxO1, mTOR, IGF-II and PI3K/Akt pathway presents a feedback loop that can preferably fine-tune the regulation of muscle differentiation. Moreover, FoxO1 can inhibit early step of myoblast differentiation through interacting with Notch signaling and promoting corepressor clearance and recruiting the coactivator of Csl, leading activation of Hes family, which is considered to be a myoblast differentiation repressor. Notably, although FoxO1 suppresses the early muscle differentiation process, FoxO1 is required for myoblast terminal differentiation fusion into myotubes. However, the molecular mechanism in which FoxO1 is required for myotube fusion has remained poorly understood.

There are plenty of researches showed that FoxO1 is widely expressed in various tissues, such as liver, fat, and skeletal muscle [27, 28], especially richly expressed in stem cells and adult skeletal muscle [27, 29]. Its function on muscle differentiation has been reported in considerable studies (Table 1). Several signals, including phosphatidylinositol 3-kinase (PI3K)/Akt pathway, insulin, insulin receptor substrate-1 (IRS-1) and Rho/ROCK signaling, have been demonstrated to mediate FoxO1 transcriptional activity through a phosphorylation-mediated nuclear exclusion event during myogenic differentiation (Figure 1).

**Table 1:** FoxO1 functions on different myoblast differentiation stage

Myoblasts mold	Functions on myoblast differentiation		Conclusion	Year	Reference
	Inhibits myoblast early differentiation	Required for myoblast fusion			
Mouse primary myoblasts		Yes	FoxO1 is required for mouse primary myoblast fusion	2003	[21]
C2C12 myoblasts	Yes		An active form of FoxO1 mutant inhibits C2C12 cell differentiation whereas an inactive mutant FoxO1 can partially restore inhibition of C2C12 cell differentiation regulated by wortmannin	2003	[33]
C2C12 myoblasts		Yes	Inactivation of Rho/ROCK signaling is crucial for myoblast fusion and nuclear translocation of FoxO1	2004	[49]
C2C12 myoblasts		Yes	Negative-feedback loop between FoxO1a and cGKI fine-tunes the progress of muscle cell fusion process	2005	[76]
C2C12 myoblasts	Yes		Interaction between FoxO1 and Notch1 inhibits myoblast differentiation through promoting corepressor clearance and recruiting the coactivator of Csl, leading activation of Hes family, which is considered to be a myoblast differentiation repressor	2007	[25]
C2C12-RasV12/C2C12-RasV12C40 myoblasts	Yes		Nuclear exclusion of FoxO1 is required for C2C12-RasV12C40 myoblast differentiation	2008	[43]
C2C12 myoblasts	Yes		FoxO1 negatively regulates myoblast differentiation through degradation of mTOR pathway components	2008	[52]
L6 myoblasts		Yes	Inhibition of FoxO1 transcriptional activity or nuclear exclusion of FoxO1 suppresses L6-mIRS1 cell differentiation	2011	[22]
C2C12 myoblasts	Yes		PAX3/FOXO1A and PAX7/FOXO1A suppress myogenesis through inhibiting transcriptional activity of MyoD-target genes	2013	[70]
Rhabdomyosarco-ma cells	Yes		PAX3-FOXO1 fusion protein inhibits rhabdomyosarcoma cell differentiation through upregulating JARID2	2014	[74]
C2C12 myoblasts	Yes		Insulin triggers FoxO1 nuclear exclusion and protein degradation to reverse inhibited myogenesis by FoxO1	2014	[35]

### Akt pathway

In established cell lines, activation of FoxO1 induces cell cycle arrest followed by apoptosis. However, activation of PI3K/Akt pathway removes the effect through phosphorylating FoxO1 [30–32]. Subsequently, phosphorylated FoxO1 was shuttled out of nucleus to the cytoplasm and lost the regulatory function. FoxO1 gain-of-function mutant inhibited myoblast differentiation in C2C12 cells and blocked myotube fusion that was induced by constitutively active Akt, whereas the dominant-negative FoxO1 led to a slight but striking increase in the expression of differentiation markers, myogenin and MyHC [33]. Moreover, the inactive form of FoxO1 can be able to partly but not completely rescue the repression of differentiation mediated by wortmannin, a specific inhibitor of PI3K/Akt pathway, to block the PI3K/Akt ability to phosphorylate FoxO1 [33, 34]. These data revealed FoxO1 as a key effector of the PI3K/Akt pathway in skeletal muscle differentiation. In addition, inactive FoxO1 cannot fully reverse the suppression of differentiation caused by wortmannin which may account for the multiple downstream effectors of PI3K/Akt pathway in differentiation process [33].

### Insulin

A study conducted by Wu et al. [35] revealed that overexpression of FoxO1 dramatically inhibited C2C12 myoblasts differentiation. Previous studies demonstrated insulin and IGF-I as crucial regulators of FoxO1 subcellular localization and intense potentiators of myogenic differentiation through a PI3K-Akt dependent way [36]. FoxO1 inhibited the generation of insulin-positive cells [37] and insulin treatment significantly restored the inhibiting myogenic differentiation caused by FoxO1 [35]. Moreover, treatment with LiCl, a strong stimulator of myogenesis and an activator of Wnt signaling that cooperates with insulin to promote myogenesis [38, 39], parallelly significantly restored FoxO1 inhibiting function on myogenic differentiation [40]. Simultaneous application of insulin and LiCl can distinctly overcome the inhibiting effect caused by active FoxO1 and promote myoblast differentiation synergistically [35, 40].

In addition, nuclear exclusion of FoxO1 blocks the transcriptional regulatory roles on its target genes [41]. FoxO1 is located in both cytoplasm and nucleus in proliferating myoblasts, while it is exported to the nucleus and no more transported into nucleus when myoblasts fused to form multinucleated myotubes. Series of studies revealed that nuclear exclusion of FoxO1 is a crucial step for early myogenic differentiation [42, 43]. Insulin dramatically reduced the FoxO1 nuclear level and the stability of FoxO1 protein [44, 45], suggesting that insulin triggered FoxO1 nuclear exclusion and protein degradation to reverse inhibited myogenesis by FoxO1. Taken together, FoxO1 suppressed muscle differentiation *via* directly reducing the expression of differentiation markers and repressing the promoter activity of myogenic genes, including MyoD and MEF2C, whereas this repression could be fully removed by LiCl and insulin [35, 46]. These data fully indicated that FoxO1 transcriptional activity is suppressed by insulin and IGF signaling.

### IRS-1

On the other hand, compared to the proposition that FoxO1 negatively regulated muscle cell differentiation, conflicting observations held that FoxO1 nuclear accumulation was required for muscle cell fusion. A study conducted by Hakuno et al. [22] reported that expression of a FoxO1 dominant-negative mutant, lacking 256 N-terminus residues including Akt phosphorylation sites and transcriptional activation domain, resulted in significantly decreased myogenic marker expression including myogenin and MyHC in L6 cells, indicating that the myogenic differentiation was suppressed by this FoxO1 dominant-negative mutant form [22]. In addition, the constitutive expression of IRS-1 could inhibit myoblast fusion, accompanied with excluding FoxO1 from the cells nuclei to cytosol. This cytosolic localization was correlated with FoxO1 phosphorylation in a PI3k-Akt-dependent manner. It is noteworthy that FoxO1 is localized in the nucleus during myoblast differentiation, where it exists in active form [22]. These results suggest that inhibition of FoxO1 transcriptional activity or excluding FoxO1 from the cells nuclei is at least one of the reasons why L6-mIRS1 cell differentiation is suppressed, indicating that FoxO1 transcriptional activity is required for L6 cell fusion. Thus, a pattern in which the inhibition of myogenic differentiation is at least partially caused by FoxO1 exclusion from the nuclei by IRS-1 overexpression is speculated.

### Rho/ROCK signaling

Rho GTPases are molecular switches that modulate a variety of cytoskeleton-dependent cell functions [47]. Rho and its effector, the Rho-associated kinase ROCK, also play important roles in skeletal muscle differentiation. Both Rho and ROCK were high in proliferating myoblasts but decreased during differentiation. Several reports have showed Rho to be a negative regulator of muscle differentiation. For example, no multinucleated myotubes were observed in rat L6 myoblasts transfected with an active Rho mutant even under differentiation conditions [48]. Moreover, constitutive activation of Rho or ROCK resulted in a defect in myoblast fusion but did not abrogate the expression of early differentiation markers, MyoD and myogenin [49], in association with FoxO1 cytoplasmic retention. In addition, inactivation of ROCK was required for the nuclear accumulation of FoxO1 before the onset of myoblast terminal differentiation and then highly promoted myoblast fusion. This result is further supported by observations that FoxO1 is localized in the nucleus during myoblast terminal differentiation [21, 22]. Thus, these observations are abundantly revealed that Rho and ROCK may negatively regulate myoblast fusion but not the earlier steps of differentiation and the nuclear accumulation of FoxO1 is required for myoblast fusion. Notably, FoxO1 is a direct substrate of ROCK and ROCK directly phosphorylates FoxO1 in C2C12 cells, leading the FoxO1 shuttled out of nucleus. Thus, it appears to be that down-regulation of Rho/ROCK signaling is essential for FoxO1 nuclear translocation and myoblast fusion *in vitro*, providing a novel regulatory role of Rho/ROCK signaling regulating FoxO1 localization in myogenic differentiation.

## MECHANISMS OF FOXO1 IN THE REGULATION OF SKELETAL MUSCLE DIFFERENTIATION

Although above researches believe FoxO1 is crucial for terminal myogenic differentiation, more studies mentioned below support FoxO1 as a negative regulator of muscle cell differentiation at early stage (Table 1). For example, Hribal et al. [33] showed that C2C12 myoblast differentiation was closely related to the increased FoxO1 phosphorylation and that differentiation appeared to require FoxO1 inhibition, similar to what has been revealed in thymocytes [50] and adipocytes [51]. These conflicting findings enough suggest that FoxO1 have dual roles in muscle differentiation according to the step of differentiation program (Figure 1). Here, we will declare the possible molecular pathway for FoxO1 mediating skeletal muscle differentiation.

### mTOR pathway

The result from Wu et al. [52] confirmed a FoxO1 active mutant blocked C2C12 myoblasts differentiation at an early myogenesis stage and demonstrated that a regulatory loop between FoxO1 and the mammalian target of rapamycin (mTOR) pathway in myogenic differentiation course. mTOR, a most important regulator of cellular processes including growth, survival, proliferation and differentiation, has been found to be essential for the differentiation of C2C12 myoblasts by regulating the expression of IGF-II [53–56]. The autocrine production of IGF-II, which is critically participated in skeletal muscle differentiation as well as adult muscle regeneration, is upregulated by mTOR pathway at the transcriptional level. Moreover, as a major mediator of myogenic signaling, the PI3K/AkT pathway is a downstream of IGF-II signal [53]. Since FoxO1 is a downstream target of PI3K/AkT signaling [30], the IGF-II-PI3K/Akt-FoxO1-mTOR regulatory loop appears to be a major mediator of skeletal muscle differentiation.

FoxO1 can reduce the nutrient-dependent production of IGF-II and consequently breaks the process of myogenesis that relies on the IGF-II autocrine actions through decreasing the protein levels of mTOR. Activation of an inducible mutant of FoxO1 induces proteasome-dependent degradation of mTOR pathway components that are required for differentiation, including mTOR, raptor, S6 proteinkinase 1 and tuberous sclerosis complex 2, and then attenuates IGF-II expression at the transcriptional activation level [53]. In addition, when the active FoxO1 inhibits the myocyte differentiation, treatment of exogenous IGF-II could completely rescue this inhibition of myogenesis process from FoxO1 [53]. Thus, FoxO1 appears to exert the inhibitory function on muscle differentiation by reducing the IGF-II expression through degradation of mTOR which has been identified as a critical regulator of IGF-II transcription. Therefore, degradation of mTOR pathway components by FoxO1 provides a regulatory mechanism specific to the differentiation process. Overall, the relationship among FoxO1, mTOR, IGF-II and PI3K/Akt pathway presents a feedback loop that can preferably fine-tune the regulation of muscle differentiation.

### Notch pathway

The study by Kitamura and coworkers has reported that FoxO1 is required for the inhibitory effect of Notch on myoblast differentiation and the ability to control myogenesis of FoxO1 is mediated through interaction with Notch [25]. Several reports have proved that active Notch signaling inhibits C2C12 and 10T/2 myoblasts differentiation, similar to the effect of active FoxO1 on cellular differentiation, *via* suppressing MyoD transcription [25, 57–59]. In addition, FoxO1 ablation simulates Notch1 ablation in mice [15, 60]. These data suggest that FoxO1 and Notch1 not only have a certain similarity function but also may have a further corelation on muscle differentiation. Csl is a DNA-binding protein and an identified Notch downstream effector [61]. Moreover, Hes1, another prototypical effector of Notch and also a Csl downstream target gene [62], has been considered to be a myoblast differentiation repressor by suppressing MyoD transcriptional level [53]. Kitamura et al. [25] demonstrated that through promoting corepressor clearance and recruiting the coactivator of Csl, FoxO1 physically and functionally interacted with Notch, resulting in activation of Hes1, thus inhibiting the myogenic progress. In this study, the Notch1 decoy partly rescued FoxO1 inhibition of myoblast differentiation. Likewise, FoxO1 small interfering RNA (siRNA) also rescued the inhibitory effect of Notch1 on myoblast differentiation and myosin expression. In addition, the authors also found that the DNA-binding protein Csl binds to FoxO1 through FoxO1 N terminal domain interacts with Csl N terminal and then binds to a consensus sequence in the *Hes1* promoter [25, 61]. Since the *Hes1* promoter contains no forkhead binding sites, FoxO1 cannot bind to it directly but through binding Csl element of *Hes1* in differentiating C2C12 cells. Compendiously, FoxO1 functions to repress muscle differentiation *via* accompanying with constitutive binding to the Csl-binding site in the *Hes1* promoter. Active FoxO1 and Notch1 increased the promoter activity and expression of *Hes1*, respectively, while FoxO1 siRNA inhibited this increase. These dada suggest that FoxO1 is required for Csl/Notch interaction. Meanwhile, in this study, the authors also showed that both FoxO1 and Notch1 binding to *Hes1* promoter are dependent on Csl. Notch1 binding to *Hes1* promoter is dependent on FoxO1 and the myoblast differentiation inhibited by Notch1 overexpression is rescued by inhibiting FoxO1. In addition, expression analyses found that overexpression of Notch1 or FoxO1 decreased MyoD expression, while Notch1 decoy or FoxO1 siRNA partially rescued the decrease of MyoD expression [25]. Thus, the findings present a mechanism by which FoxO1-Notch-Csl converge in a synergistic manner to suppress myoblast differentiation process *in vivo*.

### Myostatin

Myostatin, a secreted factor that belongs to the transforming growth factor (TGF)-β superfamily, plays an important role in modulating skeletal muscle type formation [63], cell growth and differentiation [64]. Myostatin is preferentially expressed in skeletal muscle [63]. It has been reported to be a potent negative regulator of myoblast proliferation and differentiation [64, 65]. Myostatin lost function leads to heavy muscle growth due to hyperplasia [66]. In contrast, increased expression of myostatin results in cachectic muscle wasting [67]. A previous study revealed that myostatin suppressed myoblast differentiation through down-regulating expression and activity of MyoD [64], whereas MyoD expression were increased in myostatin^-/-^ mice muscle. Allen et al. [68] demonstrated that myostatin is an additional target of FoxO1, since FoxO1 can directly bind and increase the activity of *myostatin* promoter to upregulate myostatin mRNA expression. Treatment of TGF-β greatly potentiated FoxO1-mediated inhibition of myoblast differentiation [68]. Together, these results suggest that FoxO1 could repress myogenic differentiation through stimulating the expression of myostatin.

### MyoD

Liu et al. [69] revealed that blocking FoxO1 expression through RNA oligonucleotide increased the myogenic factor MyoD level and skeletal muscle mass of the mice while decreased expression of the muscle negative regulator myostatin both in C2C12 cell line and *in vivo* models. The chimeric transcription factors PAX3/FOXO1 and PAX7/FOXO1 suppress myogenic differentiation, similar to the effect of dominant-negative versions of Pax3 or Pax7 constructs on terminal differentiation of satellite cells, through inhibiting transcriptional activation of MyoD target genes, including myogenin, muscle creatine kinase (MCK) and p21 while the transcriptional activity of MyoD is not perturbed [70]. Moreover, silencing the PAX3-FOXO1 fusion gene promotes myogenic differentiation in Rhabdomyosarcomas (RMS) cell lines [71]. Furthermore, transgenic PAX3-FOXO1 in mice disrupts normal myogenesis in the developing somites [72]. In addition, *JARID2* is required for cardiac myocytes and RMS cell lines differentiation [73, 74]. Knockdown *JARID2* results in decrease of cell proliferation and facilitates myogenic differentiation [74]. Walters et al. [74] reported that *JARID2* is a direct transcriptional target of the PAX3-FOXO1 fusion protein. Constitutive overexpression of PAX3-FOXO1 leads to an increase of both the RNA and protein levels of *JARID2*.

### cGKI

The cyclic GMP-dependent protein kinase I (cGKI), which regulates cytoskeleton remodeling by phosphorylating the vasodilator-stimulated phosphoprotein and receding its activity [75], is a demonstrated direct transcriptional target of FoxO1a [76]. Moreover, excessive cell fusion was observed in cGKI^-/-^ primary myoblasts [76]. In addition, cGKI is an identified regulator of FoxO1a activity and also promotes re-localization of FoxO1a out from the nucleus during muscle cell fusion. In muscle cell differentiation progress, FoxO1a directly activates transcription of cGKI. In turn, cGKI reduces the FoxO1a function in directing muscle cell fusion by phosphorylating FoxO1a, thus abolishes the binding ability of FoxO1a to its response elements [76]. This result suggests the negative-feedback loop between FoxO1a and cGKI fine-tunes the progress of muscle cell fusion process.

On the other hand, Bois and Grosveld [21] found that FoxO1 was mainly distributed in the cytoplasm in proliferating mouse primary myoblasts, while located in the nucleus by nuclear translocation from cytoplasm to nucleus in differentiated myoblasts through a non-Akt-dependent but phosphorylation-mediated nuclear exclusion event, suggesting that other kinases steer FoxO1a transcriptional activity during myogenic differentiation. Indeed, the above mentioned Rho-associated kinase ROCK and cGKI have been demonstrated to be mediators for FoxO1 transcriptional activity. Moreover, this result is consistent with the discovery that FoxO1 is localized in the nucleus during myoblast differentiation [22]. FoxO1 phosphorylation seems to decrease the primary myoblast terminal differentiation ability and a dominant-active dephosphorylated FoxO1 significantly upregulates the rate and extent of myotube formation [21, 22]. But expression of a dominant-negative FoxO1 mutant restricts myotube fusion [21]. Furthermore, inhibiting FoxO1 transcriptional activity suppressed myoblast fusion without affecting cell survival, whereas FoxO1 activation had no effect on apoptosis index of primary myoblast [21]. It is worth noting that these indispensable roles of FoxO1 on myotube formation is after the initiation of differentiation.

So, a question emerged in front of us. What precise functions do FoxO1 play in muscle differentiation? In the myoblast differentiation process, MyoD as the earliest marker of differentiation, activated the expression of myogenic specific genes to initiate the myogenic program. After a later stage of myogenic differentiation, myoblast fusion is started to form multinucleated myotubes and this is a critical step triggering terminal differentiation in a series of these events. Taken together with the before mentioned observations, FoxO1 is tightly and selectively associated with the myoblast differentiation, according to the step of differentiation program [21]. Also of note, FoxO1 is required for myoblast terminal differentiation fusion into myotubes, but at early or middle stage, FoxO1 inhibits differentiation process (Figure 1). Although numerous studies have elaborated the dual roles of FoxO1 in different stages of myoblast differentiation, the molecular mechanism of FoxO1 for myotube fusion remains poorly understood.

## ROLE OF FOXO1 IN SKELETAL MUSCLE FIBER TYPE SPECIFICATION

Skeletal muscle consists of heterogeneous specialized muscle fibers that differ in their biochemical and metabolic properties. It is this diversity of myofibers that enables different type of muscles to accomplish a variety of functions. The total number of muscle fibers is invariable prior to hatching or birth of animals, whereas the formation of the muscle fiber types could be regulated during muscle development and the muscle fiber type composition could fortunately be altered along the animal's life [77]. A striking characteristic of myofiber is the ability to remodel and transform from another type in response to environmental demands. The fiber-type switch occurs in a sequential reversible way: I ↔ IIa ↔ IIx/d ↔ IIb [78]. Many genes and signaling pathways have been reported to participate in the muscle fiber type specification and transition in developing embryos or adult muscles.

Most recent studies reported that FoxO1 plays a critical role in skeletal muscle type specification. The study of Kamei et al. [23] showed that transgenic mice specifically overexpressing FoxO1 in skeletal muscle using the skeletal muscle *α-actin* promoter weighed less than the wildtype control mice and significantly reduced the muscle mass and the size of both type I and type II fibers. This data suggests that FoxO1 may be implicated in the breakdown of muscle fibers. Meanwhile, it has been reported that FoxO1 transgenic overexpression in mice led to the decrease of type I fiber-related gene expression and presented a marked decrease in the number of type I fibers whereas the type II fiber isoform genes did not alter, accompanied with downregulation of muscle anti-fatigue ability [23]. It was also reported that in controlled conditions, FoxO1 was closely related to muscle fiber type distribution and expressed much higher in fast twitch fiber enriched muscles than in slow muscles [79–81]. In addition, a study conducted by Yuan et al. [79] demonstrated that endurance swimming exercise program induced a fast-to-slow fiber type transition, accompanied with a decrease of FoxO1 expression in both fast and slow muscles, indicating that this conversion may result from suppression of FoxO1 expression. Accordingly, this study revealed a constitutively active form of FoxO1 changed the muscle fiber type composition, accompanied by a slow- to fast-twitch fiber transition in C2C12 myoblast. These findings appear to show that FoxO1 may negatively regulate type I fiber formation but positively regulate type II. However, a contrasting finding conducted by Kitamura et al. [25] supported that FoxO1 conditional deletion in skeletal muscle using chimeric myogenin-FoxO1 transgenics decreased slow-twitch fibers and reduced expression of type I fiber genes while type II fiber genes increased. These data suggest that conditional ablation of FoxO1 decreased formation of myogenin-containing muscle fibers and changed fiber type distribution increasing MyoD-containing fibers, because myogenin is the predominant myogenic factor in slow fibers while MyoD in fast fibers [82]. This fiber-type switch in myogenin-FoxO1 mice could be accounted for the inhibitory effect of FoxO1 on MyoD expression [25]. When the inhibitory effect was removed, the formation of fast fibers was increased, potentially at the expense of slow fibers.

## MECHANISMS OF FOXO1 IN THE REGULATION OF SKELETAL MUSCLE FIBER TYPE SPECIFICATION

**Figure 2:**
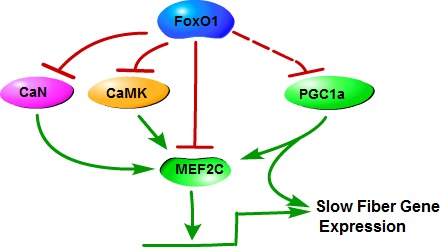
Mechanisms of FoxO1 in the regulation of slow skeletal muscle fiber gene expression FoxO1 downregulates calcineurin (CaN), CaMK and MEF2C expression, leading to a decrease of MEF2C that can increase the transcriptional activation of slow fiber genes, to inhibit slow fiber genes expression. In addition, PGC1α induces fiber-type switching from glycolytic toward oxidative fibers. FoxO1 may interact with PGC1α to inhibit certain functions of PGC1α, inhibiting expression of slow fiber gene.

FoxO1 regulates skeletal muscle fiber type specification *via* regulating the formation of slow and fast fibers through or in association with several factors and signaling pathways, such as PGC1α, MEF2C, CaMK and calcineurin pathway (Figure 2).

### PGC1α

Peroxisome proliferator-activated receptor-γ coactivator-1 (PGC1α), which co-activates PPARγ in muscle [1, 83] and is a required metabolic transcriptional coactivator for oxidative metabolism, mitochondrial biogenesis, and slow-twitch fiber formation, is a direct co-activator of FoxO1 [1, 84–86]. Several reports showed that insulin decreases the basal *PGC1α* promoter activity and Akt overexpression similar to the effect of insulin [33]. FoxO1 could directly bind to the three insulin response sequences (IRSs) addressed in the *PGC1α* promoter to upregulate the promoter activity of *PGC1α* in liver HepG2 cells [84]. However, insulin and Akt inactivate FoxO1 and inhibit the FoxO1-increased *PGC1α* promoter activity directly through phosphorylation of putative Akt sites in FoxO1, further suppressing the combining capacity of FoxO1 to *PGC1α* promoter in liver.

Previous study showed that PGC1α is abundantly expressed in skeletal muscle [87] and is remarkably induced by endurance exercise [88]. Notably, a study conducted by Lin et al. [87] showed that PGC1α is richly expressed in type I fibers and that drives the formation of slow-twitch muscle fibers. In PGC1α transgenic mice, type-II-rich muscles showed redder and better antifatigue ability than control mice and genes related mitochondrial oxidative metabolism were widely activated. Moreover, a fiber-type switching from glycolytic toward oxidative fibers was observed in transgenic PGC1α mice [87, 88]. It is widely known that type I fibers are more dependent on oxidative metabolism than type II, and this scenario may be due to the particular myofibrillar proteins and mitochondrial content much higher in type I fibers than in type II [89].

Given that PGC1α has the potential for differentiation of type I fibers and control the glycolytic to oxidative fiber-type switching, FoxO1 might be implied in these progresses. FoxO1 and PGC1α present diverse actions in different fiber type muscle. *FoxO1* mRNA level was increased in both the soleus and plantaris muscle, whereas *PGC1α* mRNA level was decreased in the soleus but not in the plantaris muscle under the condition of hindlimb unloading [90]. Furthermore, the oxidative enzyme activity and the percentage of type I fibers were reduced and the percentages of glycolytic fibers were increased in the soleus muscle, but not in the plantaris muscle during hindlimb unloading [90]. These data sufficiently suggested that both FoxO1 and PGC1α affected slow fibers more efficient than fast fibers. Regrettably, to date, we have not yet found studies verifying that FoxO1 directly physically interacts with PGC1α in skeletal muscle. But, Kamei et al. [23] showed that FoxO1 had a discrepant function with PGC1α on skeletal muscle type fiber gene: the expression of type I fiber genes, including troponin I (slow) and myoglobin, were decreased in FoxO1 transgenic mice, whereas increased in PGC1α transgenic mice. These data suggest that, although FoxO1 may facilitate PGC1α expression in muscle, decrease of type I fiber genes mediated by FoxO1 may be not directly linked to *PGC1α* gene expression, but through that FoxO1 protein interacts with PGC1α protein to inhibit certain functions of PGC1α [86]. After all, FoxO1 itself is a transcription factor.

### MEF2C and CaMK

The key regulator of muscle development, MEF2C transcription factor, is preferentially expressed in slow, oxidative myofibers and selectively active in slow oxidative fibers [91, 92]. Several reports suggest that overexpression of active form MEF2C in transgenic mice promotes slow-fiber formation and enhances running endurance through responding to calcium-dependent signaling pathway that stimulates the transformation of fast, glycolytic fibers into slow, oxidative fibers [93], whereas inactivation of MEF2C results in a severe decrease of type I fibers and losses of fiber transformation [5, 94]. Moreover, it has been conclusively suggested that MEF2C is a necessary upstream transcriptional activator of myofiber identity and troponins in skeletal muscle [95]. In addition to MEF2C, signals generated by CaMK (calmodulin-dependent kinase), downstream molecule of calcium signaling, also facilitate type I fiber gene expression. CaMK increases the transactivating function of MEF2C [92]. Although to our knowledge, FoxO1 has not been shown to be directly involved in the decision of fiber-type composition *via* MEF2C and CaMK, coincidentally enough, expression levels of both MEF2C and CaMK are significantly reduced in skeletal muscle of FoxO1 transgenic mice, indicating that the downregulation of type I fiber genes suppressed by FoxO1 may, in part, contribute to FoxO1 inhibiting the expression of MEF2C and CaMK.

### Calcineurin pathway

A study conducted Yuan et al. [79] found that overexpression of FoxO1 induced the formation of fast-twitch fibers and altered the proportion of muscle fiber type composition, along with a decrease of muscle oxidative capacity and a slow-oxidative to fast-glycolytic fiber type transformation in C2C12 myotubes. Treatment with resveratrol, which inhibited the endogenous FoxO1 activity, led to an increase of type I fiber related gene troponin (slow) and myoglobin [79] and prevented TNF-α-induced muscle atrophy [96], whereas constitutively active FoxO1 mutant significantly blocked the resveratrol-induced increased expression of these two genes. Most interestingly, addition of resveratrol could also block the FoxO1-induced slow to fast-twitch fiber transition.

It is generally known that the calcineurin pathway is a master chief regulatory pathway stimulating slow fiber-selective gene expression and slow-twitch fiber formation [97]. Several reports suggest that FoxO1 protein decreased calcineurin phosphatase activity in the cardiomyocytes [16, 98]. In turn, overexpression of calcineurin inhibited FoxO factors protein levels and precluded myotube atrophy [99]. Moreover, slow-twitch oxidative fibers present greater resistance than fast-twitch glycolytic fibers. Given the function of calcineurin on slow-twitch oxidative fiber formation and the interrelation between FoxO1 and calcineurin mentioned before, FoxO1 might be implied in calcineurin pathway controlling muscle fiber conversion. Coincidentally enough, Yuan et al. [79] revealed that a constitutively active FoxO1 mutant caused a significant decrease of endogenous calcineurin phosphatase activity and even significantly decreased the mRNA level of MCIP1.4 (modulatory calcineurin interacting protein, exon 4 isoform), a target of the calcineurin pathway. However, the mRNA level of MCIP1.4 in FoxO1-infected myoblasts was strikingly increased by resveratrol addition, suggesting that FoxO1 inhibition could reverse the negative effect on the target of calcineurin pathway expression. In summary, these results suggest that FoxO1 promotes slow-to-fast fiber-type switch and decreases muscle oxidative capacity at least, in part, through suppressing calcineurin pathway.

## CONCLUSIONS

FoxO1 is a critical transcription factor that plays an important role in skeletal muscle differentiation and fiber type specification. Although the inhibitory effect of FoxO1 on early stage of skeletal muscle differentiation by affecting several signaling including mTOR pathway, Notch pathway, myostatin, MyoD and cGKI has been known, the precise mechanisms on how FoxO1 is critical for myoblast fusion into myotube remain largely unknown. Moreover, FoxO1 is closely related to muscle fiber type specification. FoxO1 may negatively regulate type I fiber formation through inhibiting expression of MEF2C, CaMK and calcineurin or through suppressing certain functions of PGC1α, thus controlling the specification of muscle fiber type. This review has highlighted molecular mechanisms of FoxO1 in the regulation of skeletal muscle differentiation and fiber type specification. An understanding in molecular mechanisms of FoxO1 in muscle may develop new therapeutic approaches that can be used to prevent myopathies, such as muscle atrophy, in spite of great challenges that remain to be conquered.
